# Cathodoluminescence spectra of gallium nitride nanorods

**DOI:** 10.1186/1556-276X-6-631

**Published:** 2011-12-14

**Authors:** Chia-Chang Tsai, Guan-Hua Li, Yuan-Ting Lin, Ching-Wen Chang, Paritosh Wadekar, Quark Yung-Sung Chen, Lorenzo Rigutti, Maria Tchernycheva, François Henri Julien, Li-Wei Tu

**Affiliations:** 1 Department of Physics and Center for Nanoscience and Nanotechnology, National Sun Yat-Sen University, Kaohsiung, Taiwan, 80424, Republic of China; 2 Institut d'Electronique Fondamentale, UMR 8622 CNRS, University Paris Sud XI, Orsay Cedex, 91405, France

**Keywords:** gallium nitride, nanorod, cathodoluminescence, scanning electron microscopy

## Abstract

Gallium nitride [GaN] nanorods grown on a Si(111) substrate at 720°C via plasma-assisted molecular beam epitaxy were studied by field-emission electron microscopy and cathodoluminescence [CL]. The surface topography and optical properties of the GaN nanorod cluster and single GaN nanorod were measured and discussed. The defect-related CL spectra of GaN nanorods and their dependence on temperature were investigated. The CL spectra along the length of the individual GaN nanorod were also studied. The results reveal that the 3.2-eV peak comes from the structural defect at the interface between the GaN nanorod and Si substrate. The surface state emission of the single GaN nanorod is stronger as the diameter of the GaN nanorod becomes smaller due to an increased surface-to-volume ratio.

## Introduction

Recently, the applications of semiconductor materials in optoelectronic devices grow rapidly. Among them, due to the high thermal conductivity, wide direct bandgap, and chemical stability, III-V family nitride-based semiconductors, including aluminum nitride [AlN], gallium nitride [GaN], and indium nitride [InN], and their alloys have attracted lots of studies in the applications on light-emitting diodes and laser diodes. The bandgaps for AlN, GaN, and InN are 6.2 eV, 3.4 eV, and 0.65 eV, respectively. By varying the composition of these three nitride-based materials, the emission light energy will range from 0.65 eV to 6.2 eV [1]. Through the studies of the fundamental properties of the nitride-based materials, one can get more insight into the applications of these materials.

Semiconductor nanowires have attracted a lot of attention due to the large surface-to-volume ratio in the nanoscale dimension and their applications on nanodevices [2,3]. Since the first investigations of GaN nanorods (also called nanowires or nanocolumns) in 1997 [4,5], these one-dimensional [1D] GaN nanorods have attracted a lot of studies on the growth methods [6-9], physical properties [10-13], and their applications [14-16]. The geometric structures will greatly affect the optical and electrical properties of these nitride-based materials. It has been reported that 1D GaN nanorods have higher photoluminescence intensity than two-dimensional GaN due to the large surface-to-volume ratio [16]. Furthermore, in the applications of GaN materials, the defects of GaN will affect the electrical and optical properties of the GaN-based devices greatly and thus affect the performance and reliability of the devices [17].

In this work, we studied the surface topography and optical properties of the GaN nanorod cluster and single GaN nanorod via field-emission scanning electron microscopy [FE-SEM] and cathodoluminescence [CL]. The vertically aligned GaN nanorods were grown on a Si(111) substrate at 720°C without a buffer layer via plasma-assisted molecular beam epitaxy [PAMBE] [18,19]. Temperature-dependent CL spectra of the GaN nanorods were carried out to study the defect states of GaN nanorods. CL spectra at different positions along the length of the nanorod were also measured to investigate the size-dependent properties of GaN nanorods.

### Experiment

#### GaN nanorod growth

The PAMBE system used in the GaN nanorod growth was Veeco EPI 930 (Veeco Instruments Inc., Plainview, NY, USA). Ultra-high pure nitrogen gas (99.9999% purity) was supplied for the radio-frequency plasma source via a mass flow controller. The Ga source (99.999995% purity) was loaded in a Knudsen effusion cell. The base pressure of the PAMBE chamber was pumped down below 3 × 10^-11 ^Torr by a cryogenic pump. Before starting the growth process of the GaN nanorods, the Si substrate was cleaned by acetone, isopropanol, and deionized water, respectively, with ultrasonication to remove residual surface contamination. Then, the native oxide of the Si substrate was removed by a diluted hydrofluoric acid [HF] solution (HF:H_2_O = 1:5) for 5 min. The hydrogen-terminated Si(111) substrate was then transferred to the growth chamber. Prior to the growth of the GaN nanorods, the Si substrate was further annealed at 900°C for 30 min to remove atomic hydrogen [20] and residual native oxide [21] with an orderly 7 × 7 reflection high-energy electron diffraction pattern. Thereafter, the substrate was cooled down to the growth temperature of 720°C to adjust the beam equivalent pressure [BEP]. When Ga BEP was well-controlled to about 2.5 × 10^-7 ^Torr by changing the temperature of the Knudsen effusion cell, N BEP was then adjusted to about 2.5 × 10^-5 ^Torr. By preserving the growth temperature and the BEPs (Ga and N) for 3 h, the GaN nanorod cluster was successfully grown on the Si substrate.

### Separation and position of a single GaN nanorod

In order to separate and position a single GaN nanorod for CL measurement, the nanorods were scratched from the as-grown GaN nanorod substrate by a small tweezer and then knocked down on a Si substrate with a little hammer. GaN nanorods were dissolved in ethanol with an ultrasonicator for 10 min. Thereafter, the GaN nanorods were dropped on a gold-coated Si substrate covered with a copper-based network for identifying the position of the GaN nanorods.

### FESEM and CL measurement systems

The measurement system used in this work is FE-SEM (JEOL JSM-7000F, JEOL Ltd., Akishima, Tokyo, Japan). The best resolution can be approximately 1.5 nm at an acceleration voltage of 35 kV. The CL measurement was performed by the JSM-7000F FE-SEM (JEOL Ltd., Akishima, Tokyo, Japan) equipped with a Gatan MonoCL system (Gatan, Inc., Pleasanton, CA, USA). The spectrum range of the CL measurement was 200 nm to 2,300 nm.

## Results and discussion

**Figure 1:**
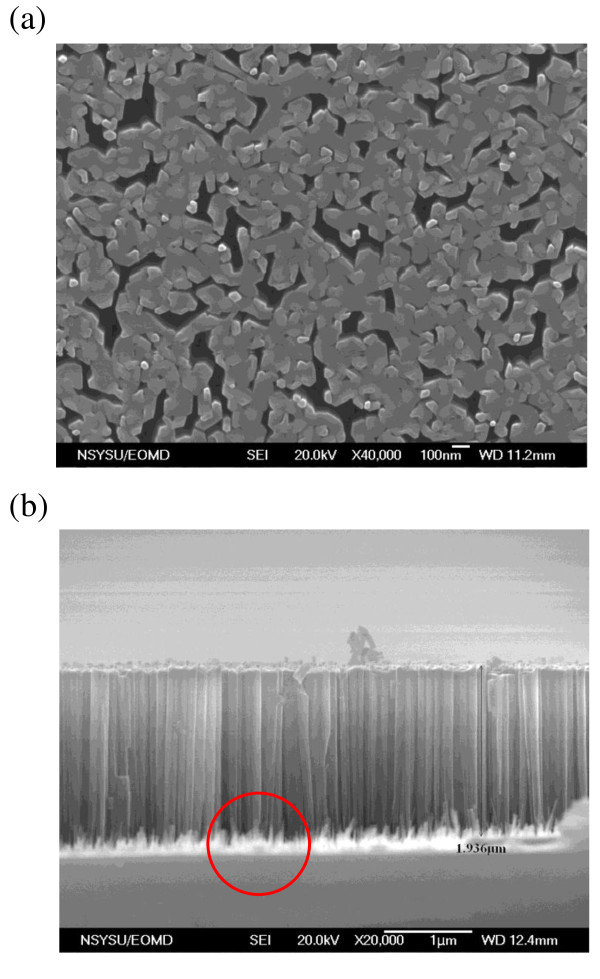
**FESEM images of GaN nanorods grown on Si(111) substrate**. (**a**) Top view and (**b**) side view.

Figure 1a, b shows the top view and the side view of the secondary electron images [SEIs] of the as-grown GaN nanorod cluster on the Si(111) substrate obtained by FE-SEM, respectively. The electron acceleration voltage is 20 kV and the magnifications of the top-view SEI and side-view SEI are ×40,000 and ×20,000, respectively. From the top-view SEI of the GaN nanorod cluster, the diameters of the nanorods on the Si substrate are about 50 to approximately 100 nm. The length of the GaN nanorods among the cluster is about 1.9 μm. There are disordered GaN nanorods appearing at the junction between the GaN nanorods and silicon substrate indicated in Figure 1b.

**Figure 2:**
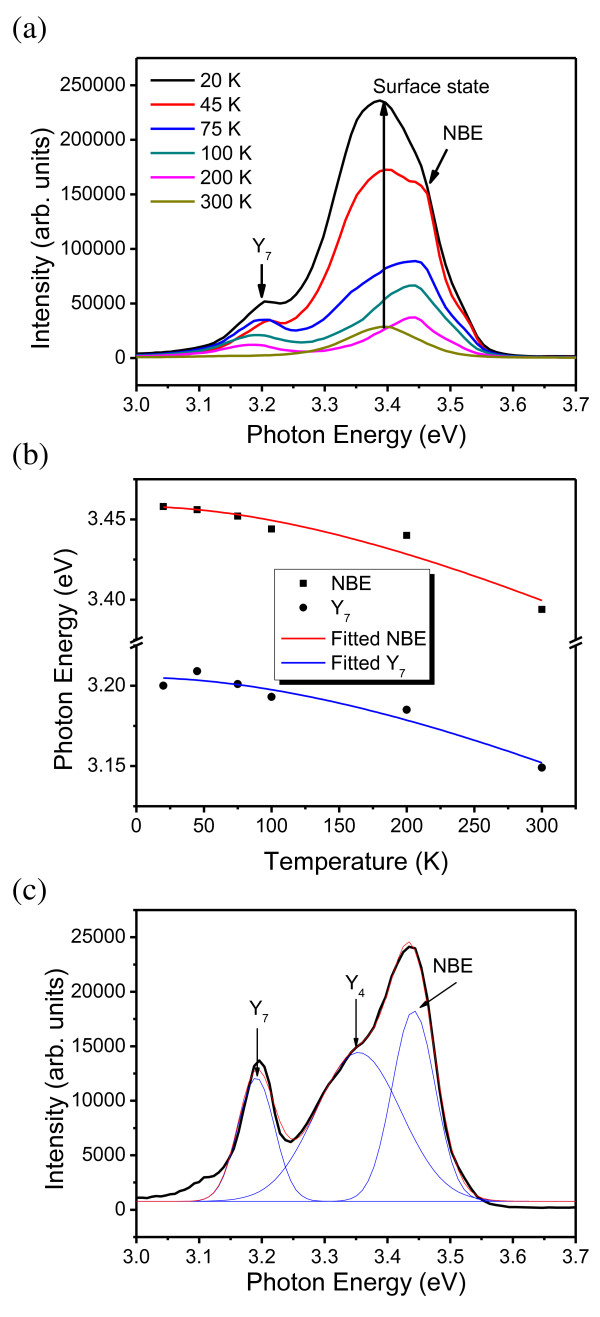
**CL spectra and peak energies**. (**a**) CL spectra of the GaN nanorods (as shown in Figure 1a) taken at temperatures of 20 K to 300 K. (**b**) Temperature-dependent NBE peak energy and defect-related (Y_7_) peak energy. The red and blue solid curves are the Varshni-equation-fitted curves of NBE and Y_7 _states, respectively. (**c**) The CL spectrum of the GaN nanorods was performed at a temperature of 20 K and at the position of the red-circled region indicated in (a). The CL spectrum was fitted by a three-peak Gaussian model.

The temperature-dependent CL spectra of the GaN nanorod cluster are measured at temperature *T *= 20 K to 300 K as shown in Figure 2a. The near-band-edge [NBE] emission of approximately 3.4 eV at *T *= 300 K reveals a blueshift with decreasing temperature as shown in Figure 2b. At *T *= 20 K, the peak energy of the NBE emission is about 3.45 eV. The peak energy change of the CL spectra for the decrease in temperature from 300 K to 20 K is 64 meV. When the temperature is lower than 100 K, the intensity of the CL spectra at 3.4 eV became stronger. The position of the peak appearing at 3.4 eV does not change with temperature, and it is ascribed to the surface state of the GaN nanorods [18] due to the low-dimensional structures of the GaN nanorods which can trap charge carriers. With the increasing temperature, the trapped charge carriers on surface states will become more unstable and thus reduce the intensity of the CL spectra. Furthermore, a peak at a photon energy of about 3.2 eV appeared at *T *= 20 K and became stronger with decreasing temperature. This peak is very weak at 300 K which cannot be easily recognized in the scale in Figure 2a. This peak corresponds to the defect state Y_7 _reported previously [17,22]. It is suggested that the Y_7 _peak comes from the recombination of an exciton bound to the point defect which is trapped by the stress field of the dislocation [17]. The temperature-dependent plots for the NBE and Y_7 _states are shown in Figure 2b. The results can be fitted with the Varshini equation [23]:

(1) $$E_{\text{g}}\left(T\right)=E_{\text{g}}\left(0\right)-\frac{\alpha T^{2}}{T+\beta },$$

where *E*_g_(*T*) is the energy gap of the semiconductor at temperature *T*, *E*_g_(0) is the energy gap at *T *= 0 K, *α *is Varshni's thermal coefficient, and *β *is the Debye temperature. The red and blue solid lines are obtained via least-square fitting according to the Varshni equation. The data were fitted well with a fixed parameter *α *= 5.3 × 10^-4 ^eV K^-1 ^because the thermal coefficient should be similar to that of the same materials grown in the same condition, e.g., GaN nanorods grown on the Si(111) substrate via the PAMBE system [24]. The fitted results for NBE state are *E*_g_(0) = 3.46 eV and *β *= 515.7 K. As to the Y_7 _state, the results are *E*_g_(0) = 3.12 eV and *β *= 598.6 K. To further investigate the source of the Y_7 _defect state, the CL spectrum at *T *= 20 K (Figure 2c) is carried out at the junction between the GaN nanorods and Si substrate as indicated in a red circle in Figure 1b. The CL spectrum is fitted with a multiple-peak Gaussian model:

(2) $$y=y_{0}+\overset{n}{\underset{i=1}{\sum }}\frac{A_{i}}{\sqrt[{w_{i}}]{\frac{\pi }{2}}}e^{-\frac{2{\left(x-x_{i}\right)}^{2}}{w_{i}^{2}}},$$

where y_0 _is baseline offset, *A_i _*is the area under each Gaussian curve from the baseline, *x_i _*is the center of each Gaussian peak, *n *= 3 is the number of peak, and *w_i _*is approximately 0.849 of the full width at half maximum for each peak. There exist three peaks of the photon energy at 3.21 eV, 3.35 eV, and 3.45 eV which correspond to Y_7_, Y_4_, and NBE, respectively. The 3.35 eV or Y_4 _peak observed in GaN has been assigned to the excitons bound to the stacking faults in the as-grown GaN samples [17]. Additionally, the Y_4 _and Y_7 _lines are reported to simultaneously appear among the GaN epilayers. For low-temperature CL measurements (*T *= 20 K), we can compare the CL spectra performed at different locations: the top of the GaN nanorod cluster and the side of the GaN nanorod cluster. The results show that the intensity ratio of the Y_7_/NBE as shown in Figure 2c became larger than that measured in the GaN nanorod cluster (shown in Figure 2a). Accordingly, we could suggest that the Y_7 _line arises from the junction between the GaN nanorods and Si substrate because the junction contains more defects, owing to the broken GaN nanorods or randomly aligned short GaN nanorods. Furthermore, the CL spectra carried out on top of the GaN nanorod cluster show a strong surface-state peak but without the Y_4 _line. In contrast, the Y_4 _line appears in the CL spectra measured on the side of the GaN nanorod cluster. The result further reveals that the surface state is due to the tip of the GaN nanorod.

**Figure 3:**
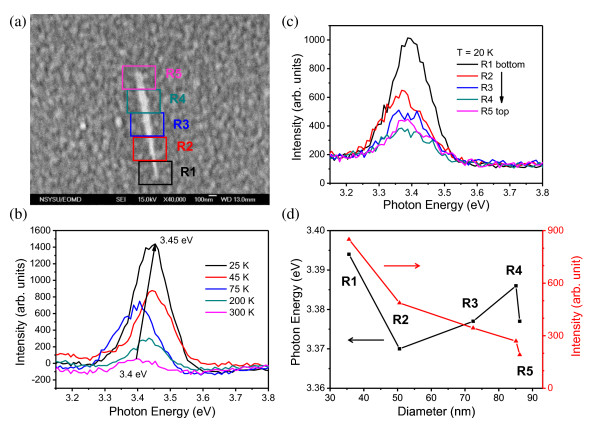
**FESEM images, CL spectra, and peak energy**. (**a**) FESEM images of a single GaN nanorod dispersed on a Si substrate. (**b**) Temperature-dependent CL spectra of a single GaN nanorod. The near-band-edge peaks were blueshifted as the temperature decreased. (**c**) Position-dependent CL spectra of the single GaN nanorod at *T *= 20 K. Each position of the GaN nanorod from top to bottom corresponds to the color box region indicated in (a). (**d**) The peak energy determined by Gaussian fitting and the peak intensity against the spectrum base line in (c) were plotted versus the GaN nanorod diameter.

Figure 3a shows the FE-SEM image of an isolated GaN nanorod placed on a gold-coated Si substrate. The length of the isolated GaN nanorod is approximately 1.3 μm which is shorter than that measured in the GaN nanorod cluster, mainly owing to the GaN isolation process. From bottom (R1) to top (R5) as indicated in Figure 3a, the diameters of the GaN nanorod which are located at the center position of each colored box are 35.6, 50.6, 72.4, 86.2, and 85.1 nm, respectively. To compare the CL spectra of the GaN nanorod cluster and that of a single GaN nanorod, the temperature-dependent CL spectra of a single GaN nanorod is measured at temperature *T *= 25 K to 300 K as shown in Figure 3b. The measured spectra exhibit fluctuation noise, mainly owing to the weakness of the CL signal of a single GaN nanorod because of the small interaction volume between the electron beam and the individual GaN nanorod. The results show a single CL peak (about 3.4 eV to 3.45 eV) at various temperatures. This peak comes from the convolution of NBE and surface state of the single GaN nanorod and also revealed a blueshift (approximately 60 meV) as temperature decreased because of the energy shift of the NBE line. In this measurement, the Y_7 _defect line is absent because the defect source of GaN could be broken and left on the Si substrate during the GaN nanorod isolation process. Furthermore, as the GaN nanorod was isolated from the Si(111) substrate which was the growth substrate and placed on the separated gold-coated Si substrate, the interface is different from that of the GaN nanorod grown on the Si(111) substrate. Additionally, the CL spectra along the GaN nanorod from bottom to top were also investigated as shown in Figure 3c. To further analyze the CL spectra of the single GaN nanorod, the CL spectra are fitted with a single Gaussian function (*n *= 1 in Equation 2). The fitted peak centers and the intensity of each peak against the base line of the CL spectra are plotted versus the GaN diameter from the bottom to the top as shown in Figure 3d. In our analysis, the peak center of the CL spectrum would be affected by the stability of the CL system and the data analysis of Gaussian fitting. In addition, the peak center will be influenced by the noise and baseline of the spectrum. Therefore, we just analyzed the peak shift between the position of the GaN nanorod at the bottom position (R1) and that at the top position (R5) measured. Compared to the top position, the CL peak shows a blueshift of about 15 meV at the bottom position. According to the quantum confinement theory developed for the Mott-Wannier type excitons of large Bohr radius (11 nm for GaN) [25] confined in nanometer-sized semiconductors, the energy shift of NBE can be expressed as [26]:

(3) $$\Delta E=\left(\frac{1}{m_{\text{e}}}+\frac{1}{m_{\text{h}}}\right)\left(\frac{h^{2}}{2D^{2}}\right),$$

where *m*_e_, *m*_h_, *h*, and *D *are the effective electron mass, effective hole mass, Plank constant, and diameter of nanorod, respectively. For GaN, *m*_e _and *m*_h _are 0.22 *m*_0 _and 1.1 *m*_0 _(*m*_0 _= 9.11 × 10^-31^kg is the electron mass) [27], respectively. The estimated Δ*E *at the bottom of the GaN nanorod (*D *≈ 35 nm) is larger than the Δ*E *at the top of GaN nanorod (*D *≈ 85 nm) by 5.3 meV which is smaller than the experimental value of 15 meV. Based on Equation 3, we can estimate that if the effective diameters of the GaN nanorod at R1 and R5 are 21 nm and 51 nm, respectively, which are about 60% of the measured values, the shifted energy will approach the experimental result of 15 meV. The reduction on the effective diameter of the GaN nanorod could be related to the band-bending effect caused by the Fermi level pinning of the GaN nanorod [28,29]. Furthermore, the peak intensity is strong as the CL spectra are carried out at the bottom of the GaN nanorod, which is mainly due to the size effects. The size effects come from the increase of the surface state density of GaN due to the large surface-to-volume ratio and the variation of electronic states because of the diameter difference. However, in CL spectra measurements, the results cannot conclude which effect dominates the increased intensity. That can be further confirmed by other measurements or experimental setups in the future.

## Conclusions

In summary, the as-growth GaN nanorod cluster and the single GaN nanorod via PAMBE growth were studied by FE-SEM and CL spectroscopy. The emissions from the NBE, surface state, Y_4 _and Y_7 _defect states of the GaN nanorod cluster, and the single GaN nanorod were investigated and analyzed. The results show that the CL spectra of the GaN nanorod cluster and the single GaN nanorod are sensitive to the change in temperature and structure of GaN. For the GaN nanorod cluster and the single GaN nanorod, the NBE line position will blueshift with the decreasing temperature, and the intensity of the CL spectra for the surface state of 3.4 eV will increase with the decreasing temperature. However, the Y_7 _defect line did not appear in the single GaN nanorod; therefore, we can deduce that the source of the Y_7 _line came from the structural defect existing between the GaN nanorods and the Si substrate. Furthermore, the position-dependent CL spectra of the single GaN nanorod revealed that the surface state of the single GaN nanorod is strongly influenced by the diameter of the GaN nanorod. These studies give us more insight in the fundamental properties of GaN nanomaterials and provide useful information in the applications of GaN nanorod-based devices.

## Competing interests

The authors declare that they have no competing interests.

## Authors' contributions

GHL carried out the SEM and CL measurements and made the initial writings. CCT gathered the data and drafted of the manuscript. YTL and CWC grew the GaN nanorod samples. PW and QYSC participated in the data analyses. LR, MT, and FHJ participated in the experimental discussions and assistance. LWT conceived this study and supervised the whole work from the experimental design and data analyses to the final version. All authors read and approved the final manuscript.
